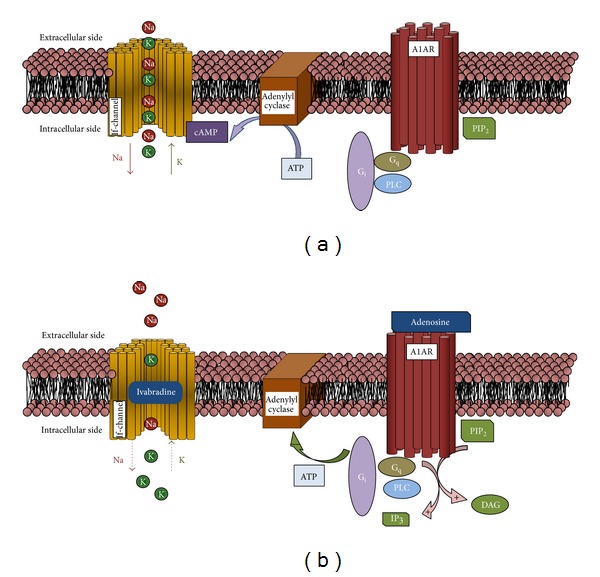# Potential Additive Effects of Ticagrelor, Ivabradine, and Carvedilol on Sinus Node

**DOI:** 10.1155/2014/932595

**Published:** 2014-09-29

**Authors:** Luigi Di Serafino, Francesco Luigi Rotolo, Augusto Boggi, Riccardo Colantonio, Roberto Serdoz, Francesco Monti

**Affiliations:** ^1^Division of Cardiology, Ospedale San Pietro Fatebenefratelli, 00189 Rome, Italy; ^2^Division of Cardiology, P.O. Di Venere, Via Ospedale di Venere, No. 1, 70131 Bari, Italy

## Abstract

A 51-year-old male patient presented to the emergency room with an anterior ST-elevation myocardial infarction. After a loading dose of both ticagrelor and aspirin, the patient underwent primary-PCI on the left anterior descending coronary artery with stent implantation. After successful revascularization, medical therapy included beta-blockers, statins, and angiotensin II receptor antagonists. Two days later, ivabradine was also administered in order to reduce heart rate at target, but the patient developed a severe symptomatic bradycardia and sinus arrest, even requiring administration of both atropine and adrenaline. Ivabradine and ticagrelor have been then suspended and this latter changed with prasugrel. Any other similar event was not reported during the following days. This clinical case raised concerns about the safety of the combination of beta-blockers and ivabradine in patients treated with ticagrelor, particularly during the acute phase of an acute coronary syndrome. These two latter drugs, in particular, might interact with the same receptor. In fact, ivabradine directly modulates the If-channel which is also modulated by the cyclic adenosine monophosphate levels. These latter have been shown to increase after ticagrelor assumption via inhibition of adenosine uptake by erythrocytes. Further studies are warrant to better clarify the safety of this association.

## 1. Background

Coronary artery disease (CAD) is the leading cause of death worldwide. Acute coronary syndromes (ACS) are responsible for a large number of cardiac-related hospital admissions. Nowadays, mortality and morbidity of patients presenting with ACS have improved as a result of the cumulative effect of multiple interventions. The management of ACS in fact has been revolutionized by contemporary therapies and strategies of care. Although coronary interventions play a critical role, medical therapy continues to retain a central place and recently several newer agents have been proposed. The purpose of this clinical case is to describe the unexpected potential additive effect of the combination of currently used drugs in the management of patients presenting with ACS.

## 2. Case Presentation

**Figure 1:**
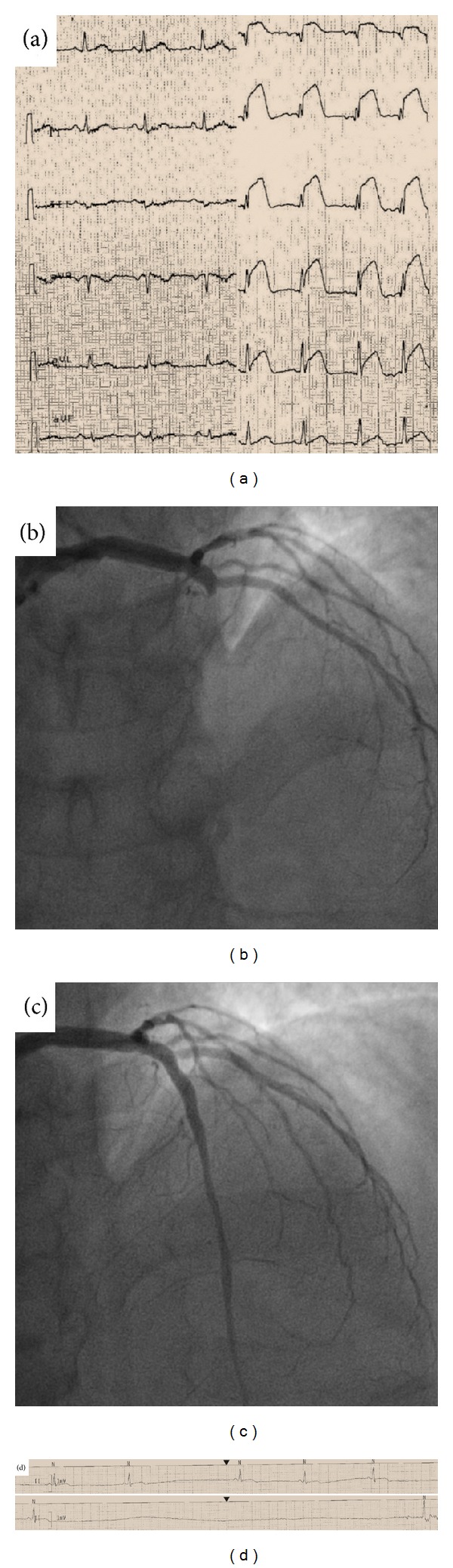


A 51-year-old male patient, with a history of hypertension and hyperlipidemia, presented to the emergency department with an anterior ST-elevation myocardial infarction (Figure 1(a)). A loading dose of both ticagrelor (180 mg) and aspirin (ASA, 250 mg i.v.) was administered and the patient sent to the cath-lab for primary percutaneous coronary intervention (PCI) on the left anterior descending coronary artery (Figures 1(b)-1(c)). After revascularization, medical therapy included beta-blockers (carvedilol 6.25 mg b.i.d.), statins (atorvastatin 80 mg), and angiotensin II receptor antagonists (telmisartan 80 mg). Dual antiplatelet therapy was continued with ticagrelor (90 mg b.i.d.) and ASA (100 mg). At the echocardiography, performed the day after the revascularization, the ejection fraction was critically reduced (EF: 25%) with akinesia of the anteroseptal wall. Titration of carvedilol was performed during the following two days without significantly reducing the heart rate at target (90 bpm with 12.5 mg carvedilol b.i.d.). Ivabradine was then administered at 5 mg b.i.d. Few hours after the first dose, the patient presented with severe symptomatic bradycardia (Figure 1(d)) and sinus arrest. Atropine and adrenaline i.v. were administered and both ivabradine and ticagrelor suspended and this latter changed with prasugrel. Any other similar event was not reported during the following days and the national competent authority was finally informed.

## 3. Discussion

High heart rate is an independent predictor of all-cause and cardiovascular mortality in coronary artery disease (CAD) patients [1]. Ivabradine, by inhibiting the sinus node “funny-current” (If), reduces heart rate without significantly affecting myocardial contractility. Normally, in cardiac pacemaker cells, If-channel is dually modulated by voltage and cyclic adenosine monophosphate (cAMP). This latter directly binds to If-channels increasing their open probability (Figure 2(a)) [2]. The safety and efficacy of ivabradine in patients with CAD have been demonstrated in several trials, and the combination with beta-blockers has also been shown to improve clinical outcome in a subset of patients [3]. However, there are no large data examining the use of ivabradine in the acute coronary syndromes and the RIVIERA study is currently expected to recruit more than 1200 patients with non-ST elevation myocardial infarction [4].

In this setting, platelet activation and subsequent aggregation play a dominant role and consequently they represent the key therapeutic targets in the management of ACS. Prasugrel and ticagrelor are currently the preferred ADP-receptor blockers since it has been shown in large trials that these drugs have higher efficacy as compared with clopidogrel [5]. In particular, in the platelet inhibition and patient outcomes (PLATO) trial, ticagrelor reduced both the composite primary endpoint (cardiovascular death, non-fatal MI, or stroke) and the cardiovascular mortality in patients with ACS [5]. However, ticagrelor may cause transient dyspnea at the onset of therapy and may also be associated with asymptomatic bradycardia in the first week of therapy [6]. Multiple hypotheses have been raised concerning the mechanism by which ticagrelor precipitates bradyarrhythmias. The first potential mechanism is that P2Y12 inhibition may directly affect cardiac conduction, but the off-target effects of ticagrelor are most likely related to the occurrence of bradyarrhythmic events. Ticagrelor, in fact, has been shown to increase adenosine concentration via inhibition of adenosine uptake by erythrocytes, probably through the inhibition of the sodium-independent equilibrative nucleoside transporters [6]. The effect of adenosine on the heart rate is well known. This is based on a direct and indirect activation of cardiac adenosine receptor 1 (A1ARs). Direct A1AR signaling effects are mediated by the activation of a potassium current through an inward rectifier potassium channel (IK_Ado_) which leads to the hyperpolarization of the sinus node cells as well as cells of the atrioventricular node (AV node) [7–9]. Indirect effects of adenosine signaling on the heart rate may involve the ability of the A1AR of lowering intracellular cAMP levels (Figure 2(b)), thereby limiting the activation of the If-channel [10]. Nevertheless, bradyarrhythmic events associated with ticagrelor have been reported as occurring most frequently in the acute phase of the ACS without any clinical consequence. By the way, the combination of ticagrelor and ivabradine has not been directly investigated and their association with beta-blockers might potentially result in clinically significant bradyarrhythmic events.

In our case, it is possible that the direct effect of ivabradine on If-channels has been unintentionally enhanced by the off-target effects of ticagrelor.

## 4. Conclusions

The above presented clinical case raised concerns about the safety of the combination of ivabradine and beta-blockers in patients treated with ticagrelor, particularly during the acute phase of an acute coronary syndrome. Further studies are warrant to better clarify the safety of this association.

## Figures and Tables

**Figure 2:**